# Isolated Medial Orbital Wall Blowout Fracture

**Published:** 2016-06-22

**Authors:** Aditya Sood, Samuel Kogan, Mark S. Granick

**Affiliations:** ^a^Division of Plastic and Reconstructive Surgery, Rutgers New Jersey Medical School, Newark; ^b^Rutgers Robert Wood Johnson Medical School, New Brunswick, NJ

**Keywords:** medial orbital wall fracture, orbital fracture, facial fractures, enophthalmos, rectus entrapment

## DESCRIPTION

**Figure 1:**
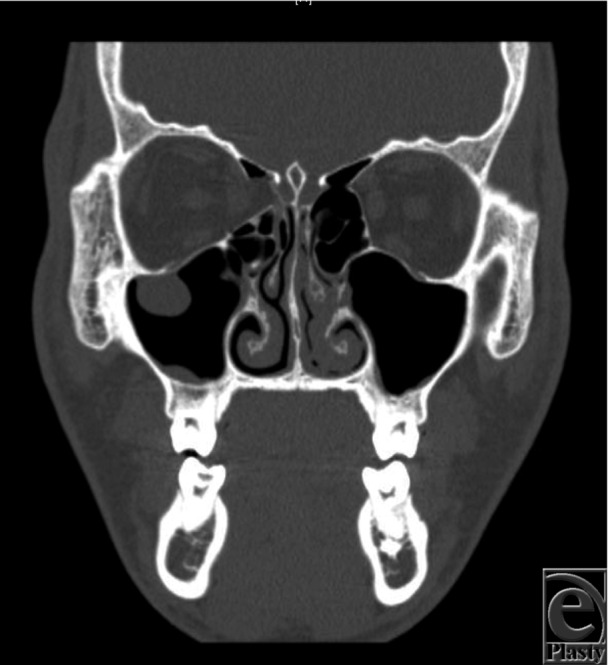
Coronal view of computed tomography max/face revealing a right-sided medial orbital wall blowout fracture without entrapment.

**Figure 2:**
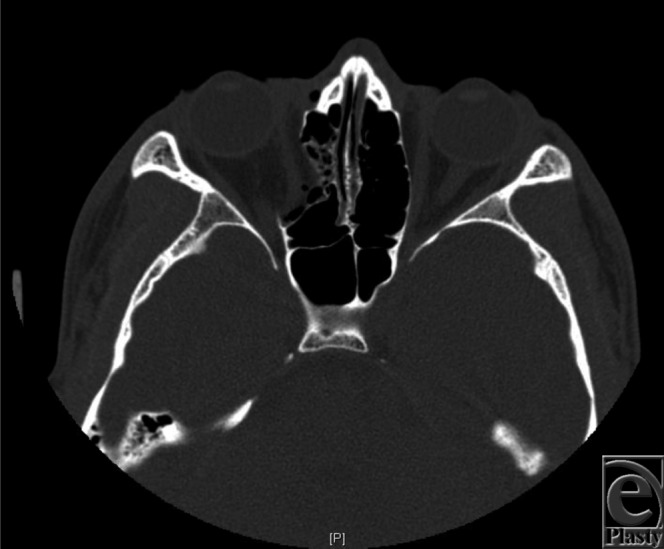
Sagittal view demonstrating similar characteristics with volume enlargement of the right orbit.

A 16-year-old adolescent boy presented to the emergency department following a closed-fist injury to his right face. Physical examination revealed swelling, ecchymosis, and subconjunctival hemorrhage of his right orbit and globe. There were no visual field disturbances. Computed tomography (CT)-radiography of his orbits revealed an isolated medial orbital wall blowout fracture without entrapment (Figs 1 and 2).

## QUESTIONS

1. **What are the etiology and prevalence of an isolated medial orbital wall blowout fracture?**
2. **What are the long-term complications of medial orbital wall fractures?**
3. **What is the classification system for medial orbital wall fractures?**
4. **What are the surgical options for medial orbital wall fractures?**

## DISCUSSION

Two competing mechanisms have been explored in the pathomechanism of medial orbital wall fractures. The buckling theory states that a force is delivered along the orbital rim, which is then absorbed and transmitted along the medial orbital wall until an area of weakness is encountered.^1^^,^^2^ Contrastingly, the hydraulic theory states that a force is applied to the globe, which is hydraulically retropulsed into the orbit and the medial orbital wall upon impact.^3^^,^^4^ With respect to all orbital fractures, the reported incidence of isolated medial wall fractures ranged from 0% to 55%.^5^ However, the incidence rises significantly when measured as part of a compound orbital floor and medial orbital wall fracture, with reported incidence of 10% to 84%.^5^

Unlike orbital floor injuries, medial orbital wall fractures are less serious, and intervention may be postponed until edema subsides.^5^ Long-term complications typically involve visual disturbances and may include medial rectus entrapment and herniation, enophthalmos, and diplopia.^5^

While no widely accepted classification system exists for medial orbital fractures, Chung et al^6^ have developed a 3-category system based on facial CT scans. Type I fractures involved a greenstick fracture in which the sinus mucoperiosteum is attached to the adjacent normal orbital wall. Type II fractures are composed of simple fractures in which the distance from anterior lacrimal crest to the deepest fracture line is less than 30 mm on CT axial view. Type III fractures involve a complex fracture, with the aforementioned distance being greater than 30 mm or having multiple fracture segments.^6^

The transcaruncular approach has become a widely accepted method for medial orbital wall reconstruction, as this method avoids a cutaneous scar.^5^^,^^6^ Preoperative CT-guided measurement of the defect size is carried out to determine appropriate implant size, with titanium mesh being the preferred implant material.^5^^,^^6^ Alternatively, a bone graft may be used, but donor site morbidity is a possibility. In some cases, a transnasal endoscopic approach may be used, with a reduction of the segments of the fractured medial orbital wall into their original position.^6^ While this method may appear ideal due to avoidance of the use of implants, repositioning of the fractured segments may occur postoperatively.^6^

In the case described earlier, this patient demonstrated significant right-sided orbital swelling upon initial examination, with no medial rectus entrapment. His defect as seen by the CT scan revealed a large type I fracture (Figs 1 and 2). Late enophthalmos may be a complication, and a delayed open reduction internal fixation with a titanium implant will be employed with a transcaruncular approach after swelling subsides.

In summary, isolated medial orbital fractures are a rare occurrence and more frequently occur in conjunction with orbital floor fractures.^1^^-^4 While the exact mechanics of injury are unknown, 2 theories have emerged. In contrast to orbital floor injuries, medial wall injuries are often less serious and rarely require immediate surgical intervention.^5^ However, medial rectus entrapment, enophthalmos, and diplopia are serious complications and warrant surgical repair of the defect.^5^ While a handful of approaches have been used, the transcaruncular approach is favored, as it is a reliable, low morbidity method, which also maintains cosmesis of the central face.^5^^,^^6^
